# A class of reduced-order models in the theory of waves and stability

**DOI:** 10.1098/rspa.2015.0703

**Published:** 2016-02

**Authors:** C. J. Chapman, S. V. Sorokin

**Affiliations:** 1 Department of Mathematics, University of Keele, Staffordshire ST5 BG, UK; 2 Department of Mechanical and Manufacturing Engineering, Aalborg University, Fibigerstraede 16, 9220 Aalborg, Denmark

**Keywords:** barycentric representation, elastic wave, Euler truncation, Fourier series, Lamb wave, Rayleigh wave

## Abstract

This paper presents a class of approximations to a type of wave field for which the dispersion relation is transcendental. The approximations have two defining characteristics: (i) they give the field shape exactly when the frequency and wavenumber lie on a grid of points in the (frequency, wavenumber) plane and (ii) the approximate dispersion relations are polynomials that pass exactly through points on this grid. Thus, the method is interpolatory in nature, but the interpolation takes place in (frequency, wavenumber) space, rather than in physical space. Full details are presented for a non-trivial example, that of antisymmetric elastic waves in a layer. The method is related to partial fraction expansions and barycentric representations of functions. An asymptotic analysis is presented, involving Stirling's approximation to the psi function, and a logarithmic correction to the polynomial dispersion relation.

## 1. Introduction

This paper is concerned with the derivation of reduced-order models for wave propagation in layered media. The starting point is a recent method [1,2] for obtaining a family of polynomial approximations to a type of transcendental dispersion relation that often arises in practice. The approximate dispersion curves intersect the exact dispersion curve, i.e. have no error, at points on a certain grid in the (frequency, wavenumber) plane; moreover, the number of points on the grid increases quadratically with the degree of the polynomial, so that the method provides accurate approximations at indefinitely large frequencies and wavenumbers.

The results in [1,2] concern the dispersion relation. The next stage, to determine the corresponding approximations to the displacement and stress fields, is the subject of this paper. The key observation is that a polynomial dispersion relation automatically reduces the problem to finite order, because it gives only finitely many modes at a given frequency (in contrast to the original transcendental dispersion relation). Hence, we seek expressions for the field variables which, likewise, are of finite order, in that they consist of finite sums of simple terms. We require that these finite sums have two properties: (i) they give the field shape exactly when the frequency and wavenumber lie on the previously found grid and (ii) they give polynomial dispersion relations.

It might be thought that such expressions for the field could not exist, in that too much is expected of them. But the authors have found that certain forms of Fourier series, if carefully chosen, give, upon truncation and application of a simple Euler correction, a family of finite approximations of the required type. Although Fourier series are the most traditional of representations in wave theory, the approach of choosing just the right series to satisfy conditions (i) and (ii), with no redundant terms, appears to be new, and is the original aspect of the present investigation. The nearest we have found in the literature to our approach is [3], which is, however, confined to low wavenumbers rather than including in its scope a large area of the (frequency, wavenumber) plane. Another analysis based on Fourier series is [4]; this is primarily a numerical approach and does not give an analytical approximation to the dispersion relation.

The structure of the paper is as follows. Section 2 gives the required theory for the example to be considered, namely antisymmetric elastic waves in a layer. Section 3 represents the displacement and stress fields by Fourier series, chosen, so that the truncations provide a family of reduced-order models of the required type, and §4 gives the corresponding barycentric representations, exact and truncated, of the dispersion relation. Section 5 reports numerical results, comparing the approximate and exact field shapes, and includes correction terms related to Euler summation. Section 6 gives a local analysis of the field shape for (frequency, wavenumber) points close to grid points, and §7 presents an asymptotic theory for the remainder term in the barycentric truncations of the dispersion relation. The asymptotic theory is based on Stirling's approximation applied to the psi function (also called the digamma function), defined as the logarithmic derivative of the gamma function. Section 8 presents conclusions, and indicates some directions for further work.

## 2. Elastic waves in a layer

### (a) Governing equations

An isotropic elastic medium of thickness *h* occupies the layer −∞<x<∞, |*y*|<*h*/2 and supports elastic waves that are assumed to satisfy the linear equations [5, pp. 59, 257]

2.1 $$u_{tt}=c_{1}^{2}u_{xx}+(c_{1}^{2}-c_{2}^{2})v_{xy}+c_{2}^{2}u_{yy}$$

and

2.2 $$v_{tt}=c_{1}^{2}v_{yy}+(c_{1}^{2}-c_{2}^{2})u_{xy}+c_{2}^{2}v_{xx}.$$

Here, (*u*(*x*,*y*,*t*),*v*(*x*,*y*,*t*)) are longitudinal and transverse displacements at position (*x*,*y*) and time *t*, and subscripts *x*, *y* and *t* denote partial differentiation. Throughout the paper, a subscript 1 refers to *P*-waves, and a subscript 2 refers to *S*-waves; thus, *c*_1_ is the *P*-wave speed, i.e. compression-wave speed, and *c*_2_ is the *S*-wave speed, i.e. shear-wave speed. The elastic medium has Young's modulus *E*, Poisson's ratio *ν*, density *ρ* and reference speed *c*_0_=(*E*/*ρ*)^1/2^. In plane strain, the wave speeds are

2.3 $$c_{1}={\left\{\frac{1-\nu }{\left(1+\nu )(1-2\nu \right)}\right\}}^{1/2}c_{0}\;\mathrm{and}\;\;c_{2}=\frac{c_{0}}{{\left\{2(1+\nu )\right\}}^{1/2}}.$$

For plane stress, the value of *c*_1_ is replaced by *c*_0_/{(1−*ν*^2^)}^1/2^. In numerical calculations, we shall take *ν*=0.3. Thus, for plane strain, we have *c*_1_=1.16*c*_0_ and *c*_2_=0.62*c*_0_, and for plane stress, we have *c*_1_=1.05*c*_0_ and *c*_2_=0.62*c*_0_. The normal stresses *τ*_*xx*_, *τ*_*yy*_ and shear stress *τ*_*xy*_ corresponding to displacements (*u*,*v*) are

2.4 $$\begin{array}{rl} \tau_{xx} & =\rho \{c_{1}^{2}u_{x}+(c_{1}^{2}-2c_{2}^{2})v_{y}\}, \end{array}$$

2.5 $$\begin{array}{rl} \tau_{yy} & =\rho \{(c_{1}^{2}-2c_{2}^{2})u_{x}+c_{1}^{2}v_{y}\} \end{array}$$

2.6 $$\begin{array}{rl} \text{and}\;\tau_{xy} & =\rho c_{2}^{2}(u_{y}+v_{x}). \end{array}$$

### (b) Antisymmetric waves

We consider antisymmetric waves in the layer, so that (*u*,*v*) are (odd, even) in the transverse coordinate *y*. These reduce to simple bending waves in the limit of low frequency and wavenumber. We seek solutions of the governing equations with real frequency *ω* and longitudinal wavenumber *k* in which all components are proportional to *e*^−i*ωt*+i*kx*^. The components are taken to be linear combinations of sin⁡ly and cos⁡ly; by (2.1) and (2.2), the transverse wavenumber *l*, which may be complex, must satisfy *l*^2^=(*ω*/*c*_1_)^2^−*k*^2^ or *l*^2^=(*ω*/*c*_2_)^2^−*k*^2^, corresponding to *P*-waves and *S*-waves.

The displacement field may be written [5]

2.7 $$u=U_{1}h\frac{i\sin L_{1}Y}{\sin\frac{1}{2}L_{1}}-V_{2}h\frac{L_{2}}{K}\frac{i\sin L_{2}Y}{\cos\frac{1}{2}L_{2}}$$

and

2.8 $$v=U_{1}h\frac{L_{1}}{K}\frac{\cos L_{1}Y}{\sin\frac{1}{2}L_{1}}+V_{2}h\frac{\cos L_{2}Y}{\cos\frac{1}{2}L_{2}}.$$

We use dimensionless variables *Y* =*y*/*h*, *K*=*kh*, and

2.9 $$L_{1}^{2}={\left(\frac{\omega h}{c_{1}}\right)}^{2}-{\left(kh\right)}^{2},\;L_{2}^{2}={\left(\frac{\omega h}{c_{2}}\right)}^{2}-{\left(kh\right)}^{2}\;\mathrm{and}\;\;L_{3}^{2}={\left(\frac{\omega h}{\sqrt{2}c_{2}}\right)}^{2}-{\left(kh\right)}^{2}.$$

For the moment, *U*_1_ and *V*_2_ are arbitrary; they are dimensionless measures of the *P*-wave part of *u* and the *S*-wave part of *v* at the boundary $Y=\frac{1}{2}$. We omit the factor *e*^−i*ωt*+i*kx*^ from all field expressions. The stresses (*τ*_*xy*_,*τ*_*yy*_) corresponding to (2.7)–(2.8) are

2.10 $$\tau_{xy}=2\rho c_{2}^{2}\left\{U_{1}L_{1}\frac{i\cos L_{1}Y}{\sin\frac{1}{2}L_{1}}-V_{2}\frac{L_{3}^{2}}{K}\frac{i\cos L_{2}Y}{\cos\frac{1}{2}L_{2}}\right\}$$

and

2.11 $$\tau_{yy}=2\rho c_{2}^{2}\left\{-U_{1}\frac{L_{3}^{2}}{K}\frac{\sin L_{1}Y}{\sin\frac{1}{2}L_{1}}-V_{2}L_{2}\frac{\sin L_{2}Y}{\cos\frac{1}{2}L_{2}}\right\}.$$

In accordance with our convention, a subscript 1 refers to quantities related to *P*-waves, and a subscript 2 refers to quantities related to *S*-waves. The dimensionless frequency used in the paper is *Ω*=*ωh*/*c*_0_.

The boundary conditions at the traction-free boundaries $Y=\pm \frac{1}{2}$ are *τ*_*xy*_=0 and *τ*_*yy*_=0, giving two homogeneous equations for the coefficients *U*_1_ and *V*_2_. Equating the determinant to zero gives a relation between *ω* and *k*, namely the dispersion relation. In the variables defined above, this is [5]

2.12 $$\frac{\left(2/L_{2})\tan(\frac{1}{2}L_{2}\right)}{\left(2/L_{1})\tan(\frac{1}{2}L_{1}\right)}=-\frac{L_{3}^{4}}{K^{2}L_{2}^{2}}.$$

The coefficients satisfy

2.13 $$\frac{U_{1}}{V_{2}}=\frac{L_{3}^{2}}{KL_{1}}\tan\frac{L_{1}}{2}=-\frac{KL_{2}}{L_{3}^{2}}\tan\frac{L_{2}}{2}.$$

Hence, displacements and stresses can be written in various ways, for example without *U*_1_ or without *V*_2_. We do this without comment in simplifying formulae.

**Figure 1.**
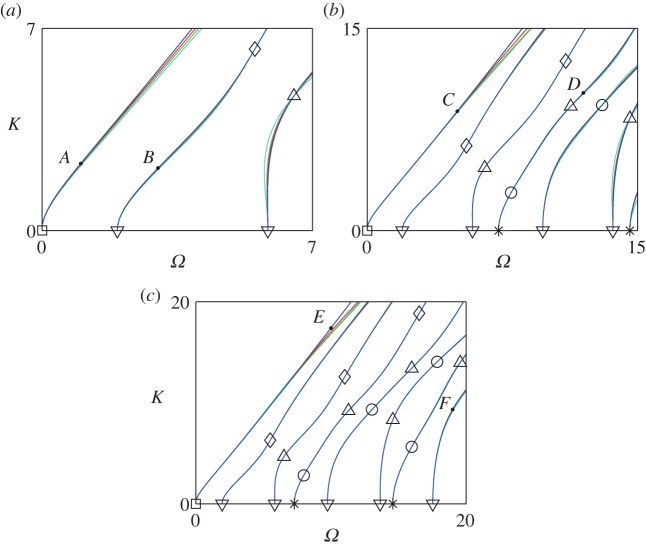
Exactand approximate dispersion relation for plane strain with Poisson's ratio *ν*=0.3. Axes are *Ω*=*ωh*/*c*_0_, *K*=*kh*; symbols are (i) Euler–Bernoulli point (square); (ii) *P*-wave cut-on points (asterisk); (iii) *S*-wave cut-on points (downside triangle); (iv) Lamé points (diamond); (v) even interior points (circles) and (vi) odd interior points (upside triangle). Exact curves are solid (blue); approximate curves, with Euler truncations of order *r*=3,2,1, are dashed (red), dashed-dotted (green) and dotted (cyan). The exact and approximate curves are indistinguishable almost everywhere; this has determined the size of the regions plotted. Values of (*m*,*n*) are (*a*) (0,1); (*b*) (2,3) and (*c*) (4,5). Field shapes for points *A*–*F*, marked with dots, are given in figure 2. (Online version in colour.)

Plots of the branches of (2.12) are given as solid curves in figure 1. The lower left corner of each plot is the Euler–Bernoulli region, describing bending waves. Grid points are marked, categorized as the Euler–Bernoulli point (square); *P*-wave cut-on points (asterisk), i.e. thickness–stretch resonances; *S*-wave cut-on points (downside triangle), i.e. thickness–shear resonances; Lamé points (diamond); even interior points (circle) and odd interior points (upside triangle). These points will be defined more fully below. Lamé and interior points are of particular interest for this work, as their significance for constructing approximations does not seem to have been recognized prior to the theory given in [1].

### (c) Reduced-order models

The exact theory just presented is unwieldy for many purposes. The underlying reason is that it includes infinitely many modes, and correspondingly gives a transcendental dispersion relation. In practice, only finitely many modes are of interest, corresponding to a range of frequencies and wavenumbers determined by the purpose of the investigation. Thus, it is usually preferable to reduce the model to finite order in some way. This paper displays explicitly a class of reduced-order models, namely those having the properties (i) and (ii) given in the introduction. That is, the reduced-order field shapes agree exactly with expressions (2.7)–(2.8) and (2.10)–(2.11) when (*Ω*,*K*) lies on the grid of points displayed in figure 1; and the approximate dispersion relations are polynomials passing through these grid points.

## 3. Fourier series representation of the field

### (a) Exact Fourier series

The transverse dependence of a field quantity may be expressed as a Fourier series in different ways, giving different periodic extensions outside the layer region $|Y|\leq \frac{1}{2}$. However, as it is usually desirable to eliminate or reduce the effects of Gibbs' phenomenon, i.e. overshoot oscillations at discontinuities, only two types of expansion are of interest, and both will be considered in this paper.

The first type of expansion is continuous. Such an expansion is obtained by requiring that all quantities are even about $Y=\frac{1}{2}$ and $Y=-\frac{1}{2}$, though discontinuities in slope are permitted at these points. For example, consider *u* and *v*. Because *u* is odd about *Y* =0 and *v* is even about *Y* =0, the extended *u* has period 2 in *Y* , i.e. 2*h* in *y*, and the extended *v* has period 1 in *Y* , i.e. *h* in *y*. This construction determines which components occur in the Fourier series of *u* and *v*, and a similar construction determines which components occur in *τ*_*xy*_ and *τ*_*yy*_. An elementary calculation, based either on the original differential equations and boundary conditions, or on (2.7)–(2.11), gives

3.1 $$\begin{array}{rl} u & =hi\frac{V_{2}}{K}\{L_{3}^{2}S(Y,L_{1})-L_{2}^{2}S(Y,L_{2})\}, \end{array}$$

3.2 $$\begin{array}{rl} v & =h\frac{U_{1}}{K}\{L_{1}^{2}C(Y,L_{1})-L_{3}^{2}C(Y,L_{2})\} \end{array}$$

and

3.3 $$\begin{array}{rl} \tau_{xy} & =2\rho c_{2}^{2}\frac{iU_{1}}{K^{2}}\{L_{1}^{2}K^{2}C(Y,L_{1})+L_{3}^{4}C(Y,L_{2})\}, \end{array}$$

3.4 $$\begin{array}{rl} \tau_{yy} & =-2\rho c_{2}^{2}\frac{V_{2}}{K^{2}}\{L_{3}^{4}S(Y,L_{1})+L_{2}^{2}K^{2}S(Y,L_{2})\}, \end{array}$$

where the functions *S* and *C* are defined by

3.5 S(Y,L)=∑n′=1∞(−1)n′sin⁡{(2n′−1)πY}14L2−{(n′−12)π}2andC(Y,L)=∑∑′m′=0∞⁡(−1)m′cos⁡(2 m′πY)14L2−(m′π)2.

A prime on a summation indicates that the first term is to be halved. Throughout the paper, *U*_1_ and *V*_2_ are related by (2.13); thus, (3.1)–(3.4) may be written with *U*_1_ and *V*_2_ interchanged, if expressions in $\tan(L_{1}/2)$ and $\tan(L_{2}/2)$ are included.

The second type of expansion is analytic up to and well beyond the layer boundaries $Y=\pm \frac{1}{2}$, but discontinuities in value or slope are permitted far enough away from these boundaries. In consequence, any Gibbs oscillations are remote from the layer itself. It is simplest to allow discontinuities at *Y* =±1, and take field quantities to have period 2 in *Y* , i.e. period 2*h* in *y*, though other choices are possible. The Fourier components are then determined, and a calculation similar to the previous one gives

3.6 $$\begin{array}{rl} u & =hi\frac{V_{2}}{K}\left\{\frac{L_{3}^{2}\sin\frac{1}{2}L_{1}}{L_{1}}\tilde{S}(Y,L_{1})-L_{2}\sin(\frac{1}{2}L_{2})\tilde{S}(Y,L_{2})\right\}, \end{array}$$

3.7 $$\begin{array}{rl} v & =h\frac{U_{1}}{K}\{L_{1}^{2}\cos(\frac{1}{2}L_{1})\tilde{C}(Y,L_{1})-L_{3}^{2}\cos(\frac{1}{2}L_{2})\tilde{C}(Y,L_{2})\} \end{array}$$

and

3.8 $$\begin{array}{rl} \tau_{xy} & =2\rho c_{2}^{2}\;\frac{iU_{1}}{K^{2}}\{L_{1}^{2}K^{2}\cos(\frac{1}{2}L_{1})\tilde{C}(Y,L_{1})+L_{3}^{4}\cos(\frac{1}{2}L_{2})\tilde{C}(Y,L_{2})\}, \end{array}$$

3.9 $$\begin{array}{rl} \tau_{yy} & =-2\rho c_{2}^{2}\;\frac{V_{2}}{K^{2}}\left\{\frac{L_{3}^{4}\sin\frac{1}{2}L_{1}}{L_{1}}\tilde{S}(Y,L_{1})+K^{2}L_{2}\sin(\frac{1}{2}L_{2})\tilde{S}(Y,L_{2})\right\}, \end{array}$$

where $\tilde{S}$ and $\tilde{C}$ are defined by $\tilde{S}={\tilde{S}}_{o}+{\tilde{S}}_{e}$ and $\tilde{C}={\tilde{C}}_{o}+{\tilde{C}}_{e}$ with

3.10 $$\begin{array}{rl} {\tilde{S}}_{o}(Y,L) & =\sum_{n^{\prime }=1}^{\infty }\frac{-(2n^{\prime }-1)\pi \sin\{(2n^{\prime }-1)\pi Y\}}{\frac{1}{4}L^{2}-{\left\{(n^{\prime }-\frac{1}{2})\pi \right\}}^{2}},\;{\tilde{S}}_{e}(Y,L)=\sum_{m^{\prime }=1}^{\infty }\frac{2\;m^{\prime }\pi \sin(2\;m^{\prime }\pi Y)}{\frac{1}{4}L^{2}-{\left(m^{\prime }\pi \right)}^{2}}, \end{array}$$

3.11 C~o(Y,L)=∑n′=1∞−cos⁡{(2n′−1)πY}14L2−{(n′−12)π}2,C~e(Y,L)=∑∑′m′=0∞⁡cos⁡(2 m′πY)14L2−(m′π)2.

The subscripts *o* and *e* indicate the odd multiples 2*n*′−1 and even multiples 2*m*′ of *πY* appearing in the trigonometric terms.

### (b) Truncated Fourier series

A decisive feature of the above Fourier series is that denominators of individual terms are zero at certain values of *L*_1_ and *L*_2_. This is a great strength of these representations, because the apparent singularities are removable, on multiplying all expressions by a factor independent of *Y* , which is permissible, because the problem is homogeneous. When this is done, only the terms which start out with zero denominators remain, because the other terms have all been multiplied by zero. Hence, zero denominators serve as labels indicating exact solutions of the problem obtainable from individual terms in the Fourier series. Two denominators can vanish simultaneously, because *L*_1_ and *L*_2_ may be chosen independently. Any such choice gives a point (*Ω*,*K*), because *L*_1_ and *L*_2_ are functions of *Ω* and *K*. Hence, the choice of a discrete set of pairs of values (*L*_1_,*L*_2_) gives a corresponding set of points in the (*Ω*,*K*) plane, i.e. a ‘grid’.

The approach just outlined may be implemented by truncating the Fourier series separately for different series. For example, one may distinguish series involving *L*_1_ from those involving *L*_2_, i.e. the *P*-wave and *S*-wave series, and use separate truncation orders, which may be varied independently as parameters. Alternatively, one may distinguish even-multiple series from odd-multiple series, as defined in (3.10)–(3.11), and truncate these at different orders. Any truncation of the Fourier series to a finite number of terms gives the field shape exactly on a grid of points in the (*Ω*,*K*) plane. When successively greater numbers of terms are retained in the truncations, the grid extends to successively larger regions, i.e. higher frequencies and wavenumbers. The truncation parameters give excellent control of the trade-off between the simplicity of an approximation and its accuracy over a wide range. A technicality in implementing the method is that the ratio *U*_1_/*V*_2_ may tend to zero or infinity as the relevant values of *L*_1_ and *L*_2_ are approached, because of the tangent terms in (2.13). Hence, a zero in one denominator may imply a zero in another, and in this case, all of the corresponding terms must be retained. This is accomplished by a simple limiting process, or the application of L'Hôpital's rule, and is carried out in §6, where a local analysis near grid points is presented.

One interpretation of the truncated Fourier series is that they represent the wave field as a set of coupled oscillators. Thus, the formulae we have obtained may be used to write down explicitly the coupled partial differential equations which correspond to the reduced-order models. Mathematically, this is equivalent to adopting Whitham's wave hierarchy approach [6, p. 353], in that the coupled equations contain products of a large number of low-order operators, namely *L*^2^−{(2*n*′−1)*π*)}^2^ and *L*^2^−(2 *m*′*π*)^2^ for *n*′=1,…,*n* and *m*′=1,…,*m*. Here, *L*^2^ is of the form given by the right-hand sides of (2.9), but with (*ω*^2^,*k*^2^) replaced by (−∂^2^/∂*t*^2^,−∂^2^/∂*x*^2^) if the space–time domain is used in preference to the frequency–wavenumber domain. The results of this paper show that there exist wave hierarchies of this type which display extremely high accuracy at only modest positions in the hierarchy. From this point of view, our results provide a generalization of the reduced-order models of the type pioneered by Mindlin and Timoshenko, and in continuous use since then [7]; until now, these models have been restricted to the lower parts of the first and second branches of the dispersion relation, and have not been extended to the higher branches.

## 4. Barycentric representation of the dispersion relation

### (a) Exact barycentric representation

When the representations in §3 are used in imposing the boundary conditions at $Y=\pm \frac{1}{2}$, and common factors are cancelled, the dispersion relation takes the form

4.1 L34K2∑n′=1∞(−1)n′(2n′−1)π14L12−{(n′−12)π}2∑∑′m′=0∞⁡(−1)m′L214L22−(m′π)2=−L1L2∑n′=1∞(−1)n′(2n′−1)π14L22−{(n′−12)π}2∑∑′m′=0∞⁡(−1)m′L114L12−(m′π)2.

This must be equivalent to (2.12), and the equivalence arises through the partial fraction expansions of $\sec(\frac{1}{2}L)$ and $\mathrm{cosec}(\frac{1}{2}L)$, which are [8, p. 118]

4.2 sec⁡L2=∑n′=1∞(−1)n′(2n′−1)π14L2−{(n′−12)π}2andcosecL2=∑∑′m′=0∞⁡(−1)m′L14L2−(m′π)2.

That is, (4.1) is obtained from (2.12) by writing the tangents as sec⁡(⋅)/cosec(⋅), and then using (4.2) to express each of $\tan(\frac{1}{2}L_{1})$ and $\tan(\frac{1}{2}L_{2})$ as the ratio of two partial fraction expansions.

In recent years, the representation of a function as a ratio of partial fraction expansions has emerged as central in approximation theory [9–11], where it is referred to as the barycentric representation. The key aspect is immediate access to the zeros of the denominators in the partial fractions, which succinctly encode the most important analytical features of the function, especially its zeros and poles. Thus, our analysis has revealed that in problems of wave propagation in a layer there is an intimate connection between Fourier series representations of the field and barycentric representations of the dispersion relation. This is the mathematical observation on which the paper is based, and is believed to be new; its exploitation will be seen to have considerable power.

### (b) Truncated barycentric representation

In (4.2), we may chose values *L*_1_ and *L*_2_ for *L*, so that two denominators vanish simultaneously, and hence obtain values of (*Ω*,*K*) for which the dispersion relation (4.1) is satisfied exactly. The resulting grid of points in the (*Ω*,*K*) plane is the same as that found in §3b, for which the Fourier series reduce to single terms. Hence, truncation of the barycentric representation (4.1) of the dispersion relation corresponds precisely to truncation of the Fourier series of the field as described in §3b, and the parameters specifying the orders of truncation in the field and in the dispersion relation also correspond. When cleared of fractions, the truncated terms in (4.1) become polynomials. Thus, truncations in (4.1) give polynomial approximations to the dispersion relation, which pass exactly through points on the grid in the (*Ω*,*K*) plane.

In determining the local behaviour of (4.1) near values of *L*_1_ or *L*_2_ where denominators vanish, a limiting process similar to that described in §3b is required. This is easily performed using local linear expansions in differentials, or L'Hôpital's rule, as carried out in §6.

### (c) Euler truncation

To specify truncations of the expressions for sec⁡(L/2) and *cosec*(*L*/2) in (4.2), we first introduce the notation

4.3 $$s_{n^{\prime }}=s_{n^{\prime }}(L)=\frac{{\left(-1\right)}^{n^{\prime }}(2n^{\prime }-1)\pi }{\frac{1}{4}L^{2}-{\left\{(n^{\prime }-\frac{1}{2})\pi \right\}}^{2}},\;c_{m^{\prime }}=c_{m^{\prime }}(L)=\frac{{\left(-1\right)}^{m^{\prime }}L}{\frac{1}{4}L^{2}-{\left(m^{\prime }\pi \right)}^{2}}$$

and

4.4 Sn=Sn(L)=∑n′=1nsn′(L),Cm=Cm(L)=∑∑′m′=0m⁡cm′(L).

For such series of alternating terms, Euler truncation gives a highly accurate family of approximations to the corresponding infinite sum. The first member of this family is obtained from the rule ‘add half the first term omitted’; the second member is obtained from the first two terms omitted, with coefficients 3/4 and 1/4 and so on to arbitrary order *r*, with appropriate coefficients [12, p. 161]. We shall use the first three members of the family, defined by

4.5 $$\begin{array}{rl} S_{n1} & =S_{n}+\frac{1}{2}s_{n+1}, \end{array}$$

4.6 $$\begin{array}{rl} S_{n2} & =S_{n}+\frac{3}{4}s_{n+1}+\frac{1}{4}s_{n+2} \end{array}$$

4.7 $$\begin{array}{rl} \text{and}\;S_{n3} & =S_{n}+\frac{7}{8}s_{n+1}+\frac{1}{2}s_{n+2}+\frac{1}{8}s_{n+3}, \end{array}$$

and similarly for *C*_*m*1_, *C*_*m*2_, and *C*_*m*3_.

In the above notation, the (*m*,*n*) approximation to the dispersion relation (4.1), with order *r* truncation, is

4.8 $$\frac{L_{3}^{4}}{K^{2}}S_{nr}(L_{1})C_{mr}(L_{2})=-L_{1}L_{2}S_{nr}(L_{2})C_{mr}(L_{1}).$$

This is equivalent to (2.12) with Euler-truncated barycentric approximations to the tangents in the form

4.9 $$\tan\frac{L_{1}}{2}≃\frac{S_{nr}(L_{1})}{C_{mr}(L_{1})}\;\mathrm{and}\;\tan\frac{L_{2}}{2}≃\frac{S_{nr}(L_{2})}{C_{mr}(L_{2})}.$$

(Recall that *S*_*nr*_ and *C*_*mr*_ refer to secant and cosecant, not sine and cosine.)

The parameters (*m*,*n*) and *r* determine a truncation of the field. Starting with the Fourier series for *u* and *v* given in §3a, the summations over *m*′ and *n*′ are continued as far as *m* and *n*, and then *r* further terms are included, for a small value of *r*, with coefficients given by (4.5)–(4.6). The end result is that we have obtained a class of reduced-order models of the type promised at the start of the paper. Our method of derivation is general: it applies to a large class of problems in the theory of waves and stability, especially in elasticity, acoustics and electromagnetism. We shall see below that the frequency range (‘bandwidth’) of these models, even for low order, is high. That is, these models satisfy a common need, for a low-order approximation which works well up to the medium-frequency range, and is not confined merely to very low frequencies. The excellent performance of these models at such higher frequencies strongly suggests that the mathematical basis of the paper, namely barycentric approximation, is faithful to the underlying analytic structure of the exact solution.

## 5. Numerical results

The numerical performance of the above-mentioned models is excellent. Their accuracy is sufficiently high that, in a large region of interest for given (*m*,*n*), the approximate and exact dispersion relations are indistinguishable on a graph, and the field shapes are accurate on a large number of the dispersion curves within this region.

Let us be precise about the term ‘region of interest’. The series truncations are expected to be accurate up to certain values of *Ω* and *K*, but not beyond. Hence, ideal numerical behaviour would have two features: (i) for a given value of (*m*,*n*), there is a box in the (*Ω*,*K*) plane within which the (*m*,*n*) model is highly accurate and (ii) the box is large even for small values of (*m*,*n*), and its size increases rapidly with *m* and *n*. We refer to such a box as a region of interest for the given value of (*m*,*n*).

The above-mentioned ideal behaviour is closely realized. Even if ‘accurate’ is defined to mean ‘almost indistinguishable on the scale of a graph’, the boxes are surprisingly large, especially for the dispersion relation. Thus, the real interest is the size of boxes, and the presentation of results will reflect this. Boxes are chosen for which visible differences between the exact and approximate dispersion relation are just starting to appear somewhere near the boundary of box, but nowhere in the interior. Thus, the size of the plots is an important aspect of what is presented, as it gives the region of interest of a given model.

In this discussion, we have taken our criterion of accuracy to be no visible difference on the scale of a graph, i.e. accuracy to two or three significant figures. This is an appropriate criterion for most modelling purposes, especially reduced-order modelling. It is also the appropriate criterion for truncated Fourier series of functions whose periodic extensions are not analytic, because higher accuracy would require a large number of terms to be retained. In different contexts, for which rapid approach to machine precision is required, one would use an expansion based on Chebyshev or Legendre polynomials. The analytical results obtained in this paper would then no longer be available.

### (a) Dispersion curves

Figure 1*a* superposes exact and approximate dispersion curves for (*m*,*n*)=(0,1), in which the approximate dispersion curves are for Euler truncations *r*=1,2,3. The box has boundaries *Ω*=7 and *K*=7. Within this box, it can be seen that the value of *r* makes rather little difference to the location of the curves, the main difference being towards the upper part of the first branch, for which the field largely consists of a Rayleigh wave at each surface of the layer. Three branches of the dispersion relation are captured accurately. Numerical tests for this and other cases showed that the choice *r*=0, i.e. blunt truncation of (4.1) without Euler correction, is not a good idea, as the range of validity in the (*Ω*,*K*) plane is greatly reduced, and moreover gives a somewhat unpredictable range of validity as *m* and *n* are varied. This is not surprising: in a series of terms of alternating sign, the minimum sensible form of truncation is to take half of the last term retained.

The striking feature of figure 1*a* is how large the box is for such small values of *m*, *n* and *r*. This arises because the polynomial dispersion curves intersect the exact transcendental dispersion curves, i.e. are exact, at the points marked square, downside triangle, triangle and diamond in the figure, which are grid points as defined in §3b. It will be recalled that the essence of our method, based on partial fraction expansions and barycentric approximations, is that the many simple denominators and their associated zeros define this grid, on which expansions collapse to single terms, and truncations become exact. For given (*m*,*n*), a definite number of grid points is captured, so that our approach is really an interpolation method in the (*Ω*,*K*) plane.

The interpolatory nature of the method explains the loss of accuracy on the higher part of the first branch. Because this part is outside the convex hull of the grid points, it relies on extrapolation rather than on interpolation, and loss of accuracy in an extrapolated region is only to be expected. Equivalently, as the branch is ascended, the transverse length scale of the corresponding Rayleigh wave ultimately becomes too small for the resolving power of a truncation at fixed (*m*,*n*).

These features of the method are confirmed in figure 1*b,c* for (*m*,*n*)=(2,3) and (4,5), both for *r*=1,2,3. The box sizes are 15×15 and 20×20. The diamonds (◊), indicating the Lamé points, lie on a straight line, the Lamé line, as explained in [1], which contains a full account of all aspects of the grid. The excellent fit somewhat above the Lamé line, even though this is an extrapolated region, arises because the slope, as well as the position, is correctly interpolated at the Lamé points. Thus, at these points, the method is performing Hermite interpolation [11, p. 86]. The other indicated grid points in the figure are the Euler–Bernoulli point (square); *P*-wave cut-on points (asterisk); *S*-wave cut-on points (upside triangle); even interior points (circles), corresponding to captured values of *m*′ in (4.1); and odd interior points (triangle), corresponding to captured values of *n*′.

In the plots, we have taken *n*=*m*+1. This is not compulsory, but related work in [1,2] gives this as the best choice, followed by *n*=*m*. Numerical tests show that the same pattern is repeated here. The explanation is not hard to find. The dispersion relation can be written equally in terms of tangents or cotangents, their reciprocals; hence, in the barycentric approximations (4.9) to tangents, the numerator and denominator should be taken to comparable accuracy. Thus, $n-\frac{1}{2}$ should be as close as possible to *m*; and because *n* and *m* are integers, this leads to *n*=*m* and *n*=*m*+1 as candidates. It so happens here that the choice *n*=*m*+1 gives slightly more accurate results.

### (b) Field shape

In §3a, we derived two types of Fourier expansion of the field. The first, which may be called the continuous type, has a continuous extension to all *Y* , and is specified by (3.1)–(3.4); the second, which may be called the remotely discontinuous type, is discontinuous at points remote from the boundaries $|Y|=\pm \frac{1}{2}$, and is specified by (3.6)–(3.9). The summation indices *m*′ and *n*′ correspond to those used in defining the truncations of the dispersion relation, so that we can write down at once the (*m*,*n*) truncation of the field corresponding to a given (*m*,*n*) truncation of the dispersion relation.

Numerical tests show that the best results, in the sense of the largest box sizes, are obtained if the continuous type of field extension is used on the first branch of the dispersion relation, i.e. the Rayleigh wave branch, but the remotely discontinuous type is used on the other branches. The reason is that, high up on the Rayleigh wave branch, the Gibbs oscillations at remote discontinuities become sufficiently great to produce unwanted oscillations even in the layer itself, i.e. far from the discontinuities. This effect grows as the Rayleigh wave branch is ascended, because the analytical continuation from the surfaces $Y=\pm \frac{1}{2}$ to the discontinuities at *Y* =±1 is in a region of exponential growth, and the growth rate is steeper (in *Y*) at higher points on the branch. The effect is not present in the interpolatory region of the (*Ω*,*K*) plane, i.e. below the Lamé line. Hence below this line, the advantage lies with a Fourier extension which is analytic at the surfaces $Y=\pm \frac{1}{2}$ and somewhat beyond, i.e. the remotely discontinuous extension as we have defined it.

**Figure 2.**
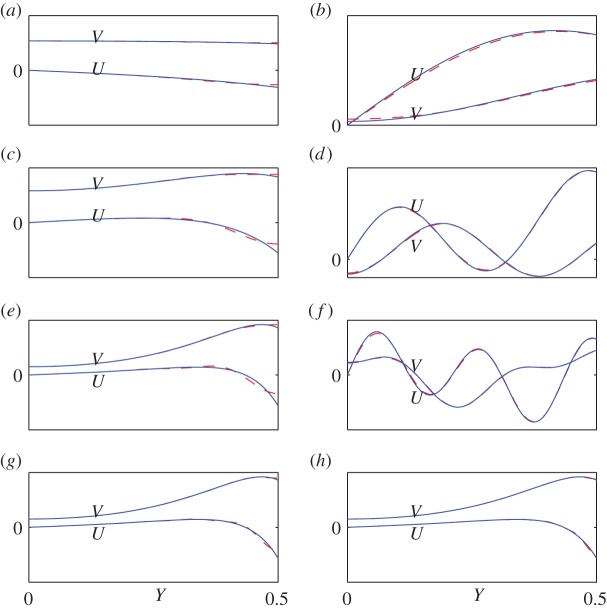
Exactand approximate field shapes (*U*,*V*) for points *A*–*F* in figure 1. Exact curves are solid (blue), and approximate curves are dashed (red). Plots (*a*–*f*) correspond to points *A*–*F*, with the same values of (*m*,*n*) as in figure 1; and plots (*g*–*h*) show further approximations, for higher (*m*,*n*), corresponding to point *E*. All plots are for Euler truncation with *r*=3, and the values of (*m*,*n*) are (*a*), (*b*) (0,1); (*c*), (*d*) (2,3); (*e*), (*f*) (4,5); (*g*) (8,9) and (*h*) (16,17). (Online version in colour.)

**Figure 3.**
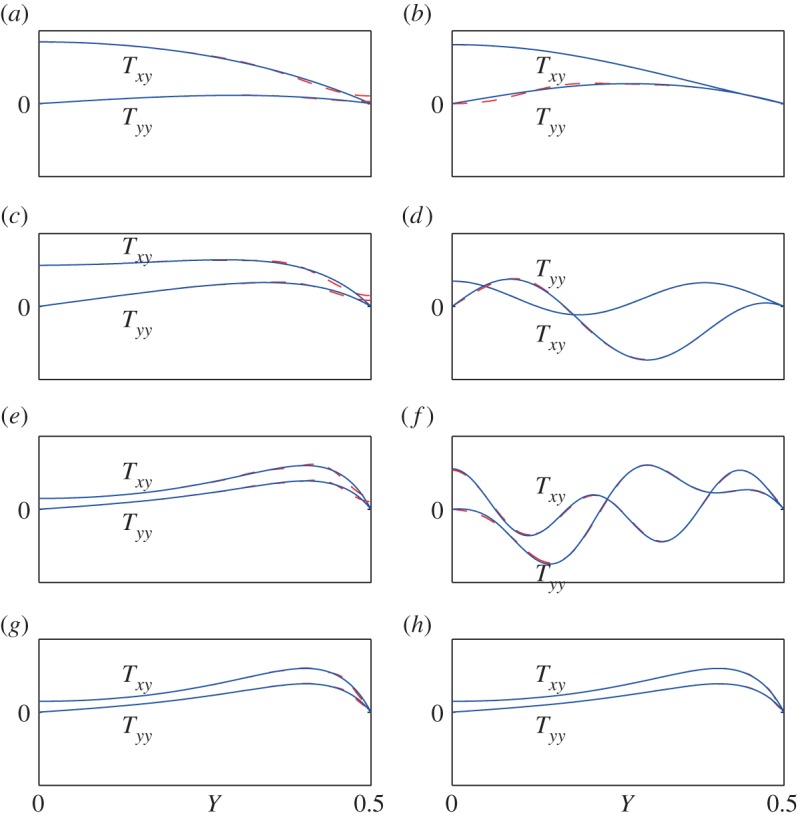
Exactand approximate stress fields (*T*_*xy*_,*T*_*yy*_) for points *A*–*F* in figure 1. Exact curves are solid (blue), and approximate curves are dashed (red). Plots (*a*–*f*) correspond to points *A*–*F*, with values of (*m*,*n*) slightly higher than in figure 2 and plots (*g*–*h*) show further approximations, for higher (*m*,*n*), corresponding to point *E*. All plots are for Euler truncation parameter *r*=3, and the values of (*m*,*n*) are (*a*), (*b*) (2,3); (*c*), (*d*), (*f*) (4,5); (*e*) (8,9); (*g*) (16,17); (*h*) (32,33). (Online version in colour.)

Figure 2 gives exact and approximate displacement field shapes (*U*,*V*) for the points *A*–*F* in the (*Ω*,*K*) plane indicated in figure 1. Here, *U* and *V* are dimensionless forms of *u* and *v* obtained by omitting the factors *h* and *i* from the right-hand side of expressions such as (2.7)–(2.8) and (3.1)–(3.2); we have also taken *V*_2_=1 and determined *U*_1_ from the coefficient relation (2.13) or its barycentric equivalent based on (4.9). Figure 3 gives the corresponding stress field shapes *T*_*xy*_ and *T*_*yy*_, i.e. the dimensionless forms of *τ*_*xy*_ and *τ*_*yy*_ obtained by omitting the factors $\rho c_{2}^{2}$ and *i* from expressions such as (2.10)–(2.11) and (3.3)–(3.4). In each figure, plots (*a*–*f*) correspond to points *A*–*F*; and (*g*) and (*h*) give higher approximations to the field corresponding to point *E*, so that (*e*), (*g*) and (*h*) form a sequence. All plots are for are for Euler truncation parameter *r*=3.

Consider first the displacements (figure 2). On the first branch, i.e. at points *A*, *C* and *E*, corresponding to plots (*a*), (*c*), (*e*), (*g*) and (*h*), the continuous type of field extension is used; for points on other branches, i.e. points *B*, *D* and *F*, which are on the second, fourth and eighth branches, corresponding to plots (*b*), (*d*) and ( *f*), the remotely discontinuous extension is used. In plots (*a*)–(*f*), the values of (*m*,*n*) correspond to those in figure 1. Thus (*m*,*n*)=(0,1), for points *A* and *B*; (*m*,*n*)=(2,3) for points *C* and *D* and (*m*,*n*)=(4,5) for points *E* and *F*. Plots (*g*) and (*h*), also for point *E*, are for parameter values (*m*,*n*)=(8,9) and (*m*,*n*)=(16,17). Thus, the plots (*e*), (*g*) and (*h*) show the effect of model order on accuracy in the most demanding region for the method.

For the stress field, the level of accuracy at given (*m*,*n*) is not quite as high as for the displacement, because of the greater steepness of the curves near the boundaries, and the values of (*m*,*n*) should be increased slightly if the same accuracy is to be maintained. This has been done in figure 3, in which the values of (*m*,*n*) are (2,3) for points *A* and *B*; (4,5) for points *C*, *D* and *F*; and (8,9), (16,17) and (32,33) for point *E*. In the last of these approximations for point *E*, the exact and approximate curves are indistinguishable on the scale of the graph.

The plots taken as a whole demonstrate the high accuracy obtainable from reduced-order models of the type proposed in this paper. Even for the low-order models, accuracy is maintained up to high frequencies and wavenumbers. Moreover, the location and magnitude of the differences between exact and approximate field shapes are easy to explain, so that the trade-off between accuracy and model order is easy to control. For example, in the first-branch approximations, for points *A*, *C* and *E*, the difference between the exact and approximate solutions is apparent only near the surfaces $|Y|=\frac{1}{2}$ of the elastic layer. This difference arises, because the continuous extension of the field, implicit in the Fourier series used, has a jump in slope at $|Y|=\pm \frac{1}{2}$. A truncated Fourier series, being analytic, must round off the region near the jump, and this is just what the plots show. For many modelling purposes, a small rounded off region is of no concern; and in any case, the sequence of plots (*e*), (*g*) and (*h*) in figures 2 and 3 shows that the rounded off region becomes almost invisibly small at model orders which are not excessively high.

## 6. Local analysis near grid points

It was pointed out in §3b that the essence of our method is the occurrence of zero denominators at carefully selected points in the (*Ω*,*K*) plane, forming the grid indicated by symbols in figure 1. Finite expressions for the field are obtained by a limiting process involving differentials. Details of this limiting process will now be presented for the displacement; the corresponding calculation for the stress is similar. We deal in turn with the even and odd interior points (circle, upside triangle), the Lamé points (diamond) and the *P*-wave and *S*-wave cut-on points (asterisk, downside triangle).

### (a) Even interior points

The even interior points (circle) are given by

6.1 $$(L_{1},L_{2})=({\tilde{L}}_{1},{\tilde{L}}_{2})=(2m_{1}\pi ,\;2m_{2}\pi ),$$

for *m*_1_=1,2,… and *m*_2_=1,2,…. The corresponding values of *Ω*, *K* and *L*_3_ are denoted $\tilde{\Omega }$, $\tilde{K}$, and ${\tilde{L}}_{3}$. Near an interior point, we take

6.2 $$(L_{1},L_{2})=({\tilde{L}}_{1}+dL_{1},\;{\tilde{L}}_{2}+dL_{2}),$$

in which *dL*_1_ an *dL*_2_ are differentials, so that only their leading powers are retained in any expression. Then, the dispersion relation (2.12) and coefficient ratios (2.13) give

6.3 $$\frac{dL_{1}}{dL_{2}}=-\frac{{\tilde{K}}^{2}{\tilde{L}}_{1}{\tilde{L}}_{2}}{{\tilde{L}}_{3}^{4}},\;\frac{U_{1}}{V_{2}}=\frac{{\tilde{L}}_{3}^{2}}{2\tilde{K}{\tilde{L}}_{1}}dL_{1}=-\frac{\tilde{K}{\tilde{L}}_{2}}{2{\tilde{L}}_{3}^{2}}dL_{2}.$$

In deriving and using (6.3), we always have available, from (6.1), the values

6.4 $$\sin\frac{1}{2}{\tilde{L}}_{1}=\sin\frac{1}{2}{\tilde{L}}_{2}=0,\;\cos\frac{1}{2}{\tilde{L}}_{1}={\left(-1\right)}^{m_{1}}\;\mathrm{and}\;\cos\frac{1}{2}{\tilde{L}}_{2}={\left(-1\right)}^{m_{2}}.$$

Hence, explicit formulae for $\tilde{\Omega }$, $\tilde{K}$ and ${\tilde{L}}_{3}$ are readily obtained from (2.9), as given in [1].

The differentials on the right-hand sides in (6.3) do not imply that terms in *U*_1_ can be ignored in expressions for *u* and *v* such as (2.7)–(2.8), because further differentials appear in denominators. The complete terms in *U*_1_ and *V*_2_ in these expressions are of the same order of magnitude; hence, $\tilde{u}$ and $\tilde{v}$ at even interior points, when expressed in terms of *V*_2_, are given by

6.5 $$\tilde{u}=V_{2}h\;i\left\{\frac{{\tilde{L}}_{3}^{2}}{\tilde{K}{\tilde{L}}_{1}}\frac{\sin{\tilde{L}}_{1}Y}{\cos\frac{1}{2}{\tilde{L}}_{1}}-\frac{{\tilde{L}}_{2}}{\tilde{K}}\frac{\sin{\tilde{L}}_{2}Y}{\cos\frac{1}{2}{\tilde{L}}_{2}}\right\}$$

and

6.6 $$\tilde{v}=V_{2}h\left\{\frac{{\tilde{L}}_{3}^{2}}{{\tilde{K}}^{2}}\frac{\cos{\tilde{L}}_{1}Y}{\cos\frac{1}{2}{\tilde{L}}_{1}}+\frac{\cos{\tilde{L}}_{2}Y}{\cos\frac{1}{2}{\tilde{L}}_{2}}\right\}.$$

These can be simplified, using (6.1) and (6.4), to give $\tilde{u}$ as a linear combination of $\sin(2m_{1}\pi Y)$ and $\sin(2m_{2}\pi Y)$, and $\tilde{v}$ as a linear combination of $\cos(2m_{1}\pi Y)$ and $\cos(2m_{2}\pi Y)$.

The terms just identified correspond to the terms with zero denominators in the Fourier series for *u* and *v* defined by (3.6)–(3.11). In this correspondence, they come from the terms with *m*′=*m*_1_ in the series for ${\tilde{S}}_{e}(Y,{\tilde{L}}_{1})$ and ${\tilde{C}}_{e}(Y,{\tilde{L}}_{1})$, and the terms with *m*′=*m*_2_ in the series for ${\tilde{S}}_{e}(Y,{\tilde{L}}_{2})$ and ${\tilde{C}}_{e}(Y,{\tilde{L}}_{2})$. No other zero denominators occur for the values of $\left({\tilde{L}}_{1},{\tilde{L}}_{2}\right)$ given by (6.1); for example, there are none in ${\tilde{S}}_{o}$ or ${\tilde{C}}_{o}$. Hence, expressions (6.5)–(6.6) are the result of applying the process described in §3b at the even interior points, parametrized by (*m*_1_,*m*_2_). The result of the process, which removes the apparent singularities, is an exact solution of the problem containing only individual terms in the Fourier series, namely (6.5)–(6.6) after simplification using (6.1) and (6.4). We have thus proved that truncations of the Fourier series (3.6)–(3.11) capture the field exactly at any desired number of the points marked by circles in dispersion diagrams as shown in figure 1.

The same argument can be applied to the other Fourier series for *v* given in §3a, defined in (3.1)–(3.2). Expression (6.6) for $\tilde{v}$ corresponds to the terms with *m*′=*m*_1_ in *C*(*Y*,*L*_1_) and *m*′=*m*_2_ in *C*(*Y*,*L*_2_), as defined in (3.5). Thus, truncations of (3.1)–(3.2) capture *v* exactly at even interior points. However, they do not capture *u* exactly at these points, because the series for *u* contains no terms of the required type in $\sin(2\;m^{\prime }\pi Y)$, but only terms in $\sin((2n^{\prime }-1)\pi Y)$. Of course, *u* is represented exactly by the infinite series of such terms, but the series does not collapse to a single term at even interior points. We shall see next that for odd interior points the situation is reversed: truncations of (3.1)–(3.2) capture *u* exactly, but not *v*. Hence, taking even and odd interior points together, there is no loss of symmetry between *u* and *v*.

### (b) Odd interior points

The field at odd interior points (upside triangle) is found in the same way, but starting with

6.7 $$({\tilde{L}}_{1},{\tilde{L}}_{2})=((2n_{1}-1)\pi ,(2n_{2}-1)\pi ),$$

for *n*_1_=1,2,… and *n*_2_=1,2,…. This gives

6.8 $$\frac{dL_{2}}{dL_{1}}=-\frac{{\tilde{K}}^{2}{\tilde{L}}_{1}{\tilde{L}}_{2}}{{\tilde{L}}_{3}^{4}},\;\frac{V_{2}}{U_{1}}=\frac{{\tilde{L}}_{3}^{2}}{2\tilde{K}{\tilde{L}}_{2}}dL_{2}=-\frac{\tilde{K}{\tilde{L}}_{1}}{2{\tilde{L}}_{3}^{2}}dL_{1}$$

and

6.9 $$\cos\frac{1}{2}{\tilde{L}}_{1}=\cos\frac{1}{2}{\tilde{L}}_{2}=0,\;\sin\frac{1}{2}{\tilde{L}}_{1}={\left(-1\right)}^{n_{1}-1}\;\mathrm{and}\;\sin\frac{1}{2}{\tilde{L}}_{2}={\left(-1\right)}^{n_{2}-1},$$

from which

6.10 $$\tilde{u}=U_{1}h\;i\left\{\frac{\sin{\tilde{L}}_{1}Y}{\sin\frac{1}{2}{\tilde{L}}_{1}}+\frac{L_{3}^{2}}{{\tilde{K}}^{2}}\frac{\sin{\tilde{L}}_{2}Y}{\sin\frac{1}{2}{\tilde{L}}_{2}}\right\}$$

and

6.11 $$\tilde{v}=U_{1}h\left\{\frac{L_{1}}{\tilde{K}}\frac{\cos{\tilde{L}}_{1}Y}{\sin\frac{1}{2}{\tilde{L}}_{1}}-\frac{{\tilde{L}}_{3}^{2}}{\tilde{K}{\tilde{L}}_{2}}\frac{\cos{\tilde{L}}_{2}Y}{\sin\frac{1}{2}{\tilde{L}}_{2}}\right\}.$$

Thus, $\tilde{u}$ is a linear combination of $\sin((2n_{1}-1)\pi Y)$ and $\sin((2n_{2}-1)\pi Y)$, and $\tilde{v}$ is a linear combination of $\cos((2n_{1}-1)\pi Y)$ and $\cos((2n_{2}-1)\pi Y)$. These correspond to the terms with *n*′=*n*_1_ in the series for ${\tilde{S}}_{o}(Y,{\tilde{L}}_{1})$ and ${\tilde{C}}_{o}(Y,{\tilde{L}}_{1})$, and *n*′=*n*_2_ in the series for ${\tilde{S}}_{o}(Y,{\tilde{L}}_{2})$ and ${\tilde{C}}_{o}(Y,{\tilde{L}}_{2})$, as defined in (3.10)–(3.11). Hence, (6.10)–(6.11), i.e. the field at odd interior points, is obtainable from individual terms in the series for *u* and *v* given by (3.6)–(3.11). Thus, truncations of the Fourier series (3.6)–(3.11) capture the field exactly at odd interior points, i.e. at any desired number of the points marked by upside triangles in dispersion diagrams as shown in figure 1.

In the alternative Fourier series for *u* and *v*, defined by (3.1)–(3.2), the trigonometric terms with argument (2*n*′−1)*πY* appear in *u* but not *v*. Hence as noted above, truncations of these series capture *u* exactly, but not *v*, and there is no loss of symmetry between *u* and *v* in our results taken as a whole. We also have an alternative explanation of a feature of the numerical results noted in §5(b). This was that in the interpolatory region of the (*Ω*,*K*) plane, i.e. within the convex hull of the symbols shown in figure 1, truncations of the Fourier series defined by (3.6)–(3.11) give more accurate representations of the field shape than do truncations of (3.1)–(3.2). The explanation is that the former is exact at more points in the (*Ω*,*K*) plane, so that the interpolation intervals are shorter. Truncations of (3.1)–(3.2) display leap-frogging, in that as a branch of the dispersion relation is ascended, either *u* or *v* is represented exactly at each symbol circle or triangle, but not both *u* and *v* at any one symbol. By contrast, truncations of (3.6)–(3.11) represent both *u* and *v* exactly each symbol.

### (c) Lamé points

The field at the Lamé points (diamond) is found by starting with $\left({\tilde{L}}_{2},{\tilde{L}}_{3})=(2\;m\pi ,0\right)$ for *m*=1,2,…, and using the corresponding values of ${\tilde{L}}_{1}$, $\tilde{\Omega }$, and $\tilde{K}$, where $\tilde{K}={\tilde{L}}_{2}=2\;m\pi$. Near a Lamé point, we take

6.12 $$(L_{2},L_{3})=({\tilde{L}}_{2}+dL_{2},{\tilde{L}}_{3}+dL_{3}).$$

This gives

6.13 $$\frac{{\left(dL_{3}\right)}^{4}}{dL_{2}}=-\frac{1}{2}{\tilde{K}}^{2}{\tilde{L}}_{1}{\tilde{L}}_{2}\cot\frac{1}{2}{\tilde{L}}_{1},\;\frac{U_{1}}{V_{2}}=-\frac{1}{2}\tilde{K}{\tilde{L}}_{2}\frac{dL_{2}}{{\left(dL_{3}\right)}^{2}}=\frac{{\left(dL_{3}\right)}^{2}}{\tilde{K}{\tilde{L}}_{1}}\tan\frac{1}{2}{\tilde{L}}_{1}$$

and

6.14 $$\sin\frac{1}{2}{\tilde{L}}_{2}=0,\;\cos\frac{1}{2}{\tilde{L}}_{2}={\left(-1\right)}^{m},$$

from which

6.15 $$\tilde{u}=-V_{2}hi\frac{\sin{\tilde{L}}_{2}Y}{\cos\frac{1}{2}{\tilde{L}}_{2}},\;\tilde{v}=V_{2}h\frac{\cos{\tilde{L}}_{2}Y}{\cos\frac{1}{2}{\tilde{L}}_{2}}.$$

Thus, Lamé points are in the same category as even interior points: truncations of the Fourier series (3.6)–(3.11) capture the corresponding field (*u*,*v*) exactly, but truncations of the series (3.1)–(3.2) capture only *v* exactly.

### (d) Cut-on points

The *P*-wave cut-on points (asterisk) are given by $\tilde{K}=0$, ${\tilde{L}}_{1}=2\;m\pi$, for *m*=1,2,…. Near a cut-on point, we take *K*=*dK*, $L_{1}={\tilde{L}}_{1}+dL_{1}$, to obtain

6.16 $$\frac{{\left(dK\right)}^{2}}{dL_{1}}=-\frac{{\tilde{L}}_{3}^{4}}{2{\tilde{L}}_{1}{\tilde{L}}_{2}}\cot\frac{1}{2}{\tilde{L}}_{2},\;\frac{U_{1}}{V_{2}}=\frac{{\tilde{L}}_{3}^{2}}{2{\tilde{L}}_{1}}\frac{dL_{1}}{dK}=-\frac{{\tilde{L}}_{2}dK}{{\tilde{L}}_{3}^{2}}\tan\frac{1}{2}{\tilde{L}}_{2},$$

and $\cos\frac{1}{2}{\tilde{L}}_{1}={\left(-1\right)}^{m}$, from which

6.17 $$\tilde{u}=V_{2}hi\frac{{\tilde{L}}_{3}^{2}}{{\tilde{L}}_{1}dK}\left\{\frac{\sin{\tilde{L}}_{1}Y}{\cos\frac{1}{2}{\tilde{L}}_{1}}-\frac{{\tilde{L}}_{1}{\tilde{L}}_{2}}{{\tilde{L}}_{3}^{2}}\frac{\sin{\tilde{L}}_{2}Y}{\cos\frac{1}{2}{\tilde{L}}_{2}}\right\}\;\mathrm{and}\;\tilde{v}=V_{2}h\frac{{\tilde{L}}_{3}^{2}}{{\left(dK\right)}^{2}}\frac{\cos{\tilde{L}}_{1}Y}{\cos\frac{1}{2}{\tilde{L}}_{1}}.$$

Here, $\tilde{u}$ is negligible compared with $\tilde{v}$, because of the squared term (*dK*)^2^ in the denominator of $\tilde{v}$, as is evident anyway from the fact that this is a thickness–stretch resonance. Thus, in examining truncations of Fourier series, we need check only for the presence of a term in cos⁡(2 mπY) in *v*. This term is present in both types of series given in §3(a); hence, truncations of both types of series capture the field shape exactly at *P*-wave cut-on points.

For *S*-wave cut-on points (downside triangle), the analysis is similar. We put $\tilde{K}=0$, ${\tilde{L}}_{2}=(2n-1)\pi$ for *n*=1,2,…, and near a cut-on point take *K*=*dK*, $L_{2}={\tilde{L}}_{2}+dL_{2}$. This gives

6.18 $$\frac{{\left(dK\right)}^{2}}{dL_{2}}=\frac{{\tilde{L}}_{3}^{4}}{2{\tilde{L}}_{1}{\tilde{L}}_{2}}\cot\frac{1}{2}{\tilde{L}}_{1},\;\frac{V_{2}}{U_{1}}=\frac{{\tilde{L}}_{3}^{2}}{2{\tilde{L}}_{2}}\frac{dL_{2}}{dK}=-\frac{{\tilde{L}}_{1}dK}{{\tilde{L}}_{3}^{2}}\cot\frac{1}{2}{\tilde{L}}_{1},$$

and $\sin\frac{1}{2}{\tilde{L}}_{2}={\left(-1\right)}^{n-1}$, from which

6.19 $$\tilde{u}=U_{1}hi\frac{{\tilde{L}}_{3}^{2}}{{\left(dK\right)}^{2}}\frac{\sin{\tilde{L}}_{2}Y}{\sin\frac{1}{2}{\tilde{L}}_{2}}\;\mathrm{and}\;\tilde{v}=U_{1}h\frac{{\tilde{L}}_{3}^{2}}{{\tilde{L}}_{2}dK}\left\{\frac{{\tilde{L}}_{1}{\tilde{L}}_{2}}{{\tilde{L}}_{3}^{2}}\frac{\cos{\tilde{L}}_{1}Y}{\sin\frac{1}{2}{\tilde{L}}_{1}}-\frac{\cos{\tilde{L}}_{2}Y}{\sin\frac{1}{2}{\tilde{L}}_{2}}\right\}.$$

Here, $\tilde{v}$ is negligible compared with $\tilde{u}$, as expected, because the wave is a thickness–shear resonance. Therefore, in Fourier series, we look for the presence of a term in sin⁡((2n−1)πY) in *u*. Such a term is present in both types of series in §3(a), and so truncations of both types of series capture the field shape exactly at *S*-wave cut-on points.

## 7. Asymptotic theory

Given the important role played in this work by barycentric approximation, it is noteworthy that exact formulae can be derived for the truncation errors incurred in using the finite sums (4.4) instead of the complete expressions (4.2) for secant and cosecant. The exact formulae, (7.8)–(7.9), appear to be new; they involve the gamma function *Γ*(*z*), especially via its logarithmic derivative, the psi function *ψ*(*z*)=*Γ*′(*z*)/*Γ*(*z*), and make available highly accurate asymptotic approximations to the remainder terms by means of Stirling's approximation. The formulae involve auxiliary functions *h*_*m*_(*z*) for $m=0,\frac{1}{2},1,\frac{3}{2},\ldots$, defined by

7.1 $$h_{m}(z)=\psi (m+1+z)-\psi (m+1-z).$$

They satisfy

7.2 $$h_{0}(z)=\frac{1}{z}-\pi \cot\pi z,$$

and the recurrence relations

7.3 $$\begin{array}{rl} h_{m}(z)-h_{m-1}(z) & =-\frac{2z}{m^{2}-z^{2}}, \end{array}$$

7.4 $$\begin{array}{rl} h_{m}(z)-h_{m}(z-1) & =\frac{2m+1}{\left(m-z+1)(m+z\right)} \end{array}$$

7.5 $$\begin{array}{rl} \text{and}\;h_{m}(z)-h_{m-1/2}\left(z+\frac{1}{2}\right) & =-\frac{1}{m-z}, \end{array}$$

obtainable from the basic identities

7.6 $$\psi (z+1)-\psi (z)=\frac{1}{z}$$

and

7.7 ψ(z)−ψ(1−z)=−πcot⁡πz

[8, ch. 5]. There are no recurrence relations giving simple expressions for *h*_*m*_(*z*)−*h*_*m*−1/2_(*z*) or $h_{m}(z)-h_{m}(z-\frac{1}{2})$.

The exact formulae giving the remainders on truncation of the series for $\sec\frac{1}{2}L$ and $\mathrm{cosec}\frac{1}{2}L$ in (4.2) are

7.8 $$\sec\frac{L}{2}=\sum_{n^{\prime }=1}^{n}\frac{{\left(-1\right)}^{n^{\prime }}(2n^{\prime }-1)\pi }{\frac{1}{4}L^{2}-{\left\{(n^{\prime }-\frac{1}{2})\pi \right\}}^{2}}+\frac{{\left(-1\right)}^{n}}{2\pi }\left\{h_{(n-1)/2}\left(\frac{L}{4\pi }+\frac{1}{4}\right)-h_{(n-1)/2}\left(\frac{L}{4\pi }-\frac{1}{4}\right)\right\}$$

and

7.9 cosecL2=∑∑′m′=0m⁡(−1)m′L14L2−(m′π)2+(−1)m−12π{hm/2(L4π)−h(m−1)/2(L4π)}.

These may be deduced from the ratio of the gamma function expressions for sine and cosine given in equations (1.2)–(1.3) of [1]; the logarithmic derivative of this ratio, after some rearrangement, gives (7.9) for the cosecant, and a transformation of the argument of (7.9) gives (7.8) for the secant.

The analytical structure of (7.8)–(7.9) is reflected in the identities (7.3)–(7.5). For example, if *m* or *n* is decreased by 1, the effect is to place the last term of the sum in the remainder; this must be equivalent to reducing the index of the remainder by 1, and this fact is expressed by (7.3). The other identities reflect periodicity with respect to *L*, and functional properties under such transformations as *L*↦*L*+2*π* or *L*↦*L*+4*π*. When written out in full, each remainder is a linear combination of four psi functions, with appropriate arguments.

Stirling's approximation to *h*_*m*_(*z*), with the $\frac{1}{12}$ correction included [8, p. 140], is

7.10 $$h_{m}(z)≃\ln\left(\frac{m+z}{m-z}\right)-\frac{z}{m^{2}-z^{2}}+\frac{z(m+1/24)}{3(m^{2}-z^{2})\{{\left(m+1/12\right)}^{2}-z^{2}\}},$$

which is highly accurate for $|z|\leq m+\frac{1}{2}$. There is a tricky point regarding the sign of the middle term in this expression. If *m* is replaced by *m*+1 in (7.3) and (7.10), the result can be rearranged to give the alternative approximation

7.11 $$h_{m}(z)≃\ln\left(\frac{m+1+z}{m+1-z}\right)+\frac{z}{{\left(m+1\right)}^{2}-z^{2}}+\frac{z(m+1+1/24)}{3({\left(m+1\right)}^{2}-z^{2})\{{\left(m+1+1/12\right)}^{2}-z^{2}\}},$$

in which the sign of the middle term has changed. At first sight, this appears to be an error. In fact, a careful re-expansion of the right-hand side of (7.11) for large *m* produces an extra term of twice the magnitude of the second term, and of opposite sign, so that the two expansions are consistent. The explanation in relation to the original infinite series is that the terms alternate in sign, and the psi functions in the definition of *h*_*m*_(*z*) are correctly keeping track of the term which comes or goes when *m* is changed by 1. Hence, either of the approximations for *h*_*m*_ may be used.

Numerical tests show that the use of approximation (7.10) or (7.11) in the barycentric form of the dispersion relation gives results of comparable accuracy to the previous results with Euler truncation of order *r*=3, which were shown in figure 1. Within the interpolation region of the figure, i.e. below the Lamé line, reasonable accuracy is maintained when some or all of the terms in (7.10) and (7.11) are omitted. However, accuracy on the Rayleigh wave branch requires at least the logarithmic term to be included, and if accuracy up to high frequencies for modest *m* and *n* is required, then all three terms should be kept.

It is a principle in asymptotic theory that at the first sign of difficulty, one should consider whether a logarithmic term has been omitted. The above results show that the principle applies here. It can happen that an expansion works well except on one branch, where a large number of terms is needed to obtain good accuracy, unless another term, of a different analytical type, is included. This phenomenon occurs in other problems in wave theory, for example in boundary-layer flows [13].

## 8. Conclusion

The results in this paper demonstrate that reduced-order models of the wave field in a layer can be accurate up to high frequencies and wavenumbers, yet correspond to dispersion relations which are only low-order polynomials. The construction of such models involves a familiar idea, that of expansion in a finite number of elementary functions, combined with an idea whose full power has only recently been appreciated, that of barycentric representation and approximation. The results provide a numerical advance on [3] to high wavenumbers, and a theoretical advance on [4] to analytical understanding of the numerical results.

As pointed out in [1], many applications of the theory of waves and stability lead to dispersion relations which, though complicated and containing many terms, amount to generalizations of (2.12) containing further trigonometric expressions. For example, they occur in problems of fluid loading [14], homogenization [15], anisotropy [16] and elastic instability [17]. The method presented in this paper applies to all problems of such type; in the case of stability analysis, the principal difference is that the frequency is allowed to be complex. Thus, reduced-order models of simple structure which are exact on a grid of points in the (frequency, wavenumber) plane can be obtained in a very wide range of problems requiring the theory of waves and stability.

## Data accessibility

The paper does not report primary data.
